# Impact of Large Aggregated Uricases and PEG Diol on Accelerated Blood Clearance of PEGylated Canine Uricase

**DOI:** 10.1371/journal.pone.0039659

**Published:** 2012-06-26

**Authors:** Chun Zhang, Kai Fan, Xuefeng Ma, Dongzhi Wei

**Affiliations:** 1 State Key Laboratory of Bioreactor Engineering, Newworld Institute of Biotechnology, East China University of Science and Technology, Shanghai, People’s Republic of China; 2 Fagen Biomedical Inc., Chongqing, People’s Republic of China; 3 Modern TCM Institute, Linyi University, Linyi, Shandong, People’s Republic of China; National Institute of Health, United States of America

## Abstract

**Background:**

Uricase has proven therapeutic value in treating hyperuricemia but sufficient reduction of its immunogenicity may be the largest obstacle to its chronic use. In this study, canine uricase was modified with 5 kDa mPEG-SPA and the impact of large aggregated uricases and cross-linked conjugates induced by difunctional PEG diol on immunogenicity was investigated.

**Methods and Findings:**

Recombinant canine uricase was first expressed and purified to homogeneity. Source 15Q anion-exchange chromatography was used to separate tetrameric and aggregated uricase prior to pegylation, while DEAE anion-exchange chromatography was used to remove Di-acid PEG (precursor of PEG diol) from unfractionated 5 kDa mPEG-propionic acid. Tetrameric and aggregated uricases were separately modified with the purified mPEG-SPA. In addition, tetrameric uricases was modified with unfractionated mPEG-SPA, resulting in three types of 5 kDa mPEG-SPA modified uricase. The conjugate size was evaluated by dynamic light scattering and transmission electron microscope. The influence of differently PEGylated uricases on pharmacokinetics and immunogenicity were evaluated in vivo. The accelerated blood clearance (ABC) phenomenon previously identified for PEGylated liposomes occurred in rats injected with PEGylated uricase aggregates. Anti-PEG IgM antibodies, rather than neutralizing antibodies, were found to mediate the ABC.

**Conclusions:**

The size of conjugates is important for triggering such phenomena and we speculate that 40–60 nm is the lower size limit that can trigger ABC. Removal of the uricase aggregates and the PEG diol contaminant and modifying with small PEG reagents enabled ABC to be successfully avoided and sufficient reduction in the immunogenicity of 5 kDa mPEG-modified tetrameric canine uricase.

## Introduction

Uricase [EC 1.7.3.3] is an enzyme involved in the purine degradation pathway, that catalyzes the oxidation of uric acid to allantoin [1]. Humans are devoid of active uricase and the accumulation of uric acid in blood causes gout symptoms [2]. Recombinant *Aspergillus flavus* uricase (Rasburicase) has been used to treat hyperuricemic disorders [3] but the potential immunogenicity limits its long-term use [4]. Pegylated uricase, with low immunogenicity and a long circulation half-life, has been under clinical investigation since the 1980s [5]. Most of the uricases used in pegylation research were from fungal origins [6] and no PEGylated microbial uricases have become available for further clinical investigation. However, a PEGylated porcine-like recombinant uricase [7] (Pegloticase) has been marketed since September 14, 2010 [8], suggesting that mammalian uricase is more suitable for developing PEGylated uricase intended for long-term use. However, 92% of patients developed antibodies and 58% of patients showed decreased urate-lowing efficacy after repeated administration during clinical trials of Pegloticase [9], [10], [11]. The largest obstacle to clinical use may lie in the sufficient reduction of the immunogenicity of PEGylated uricases.

Active uricase from all species is a homo-tetramer [12], in which one-third of the residues are hydrophobic [13], with a tendency for the tetramers to aggregate to form octomers or larger aggregates. The high molecular weight aggregates are highly immunogenic but their contents cannot be judged by SDS-PAGE or reversed phase-high performance liquid chromatography (RP-HPLC), which can only evaluate the homogeneity of denatured monomeric uricase. The native aggregated uricase content is important but has been ignored in most published papers [6], [14], [15].

**Figure 1:**
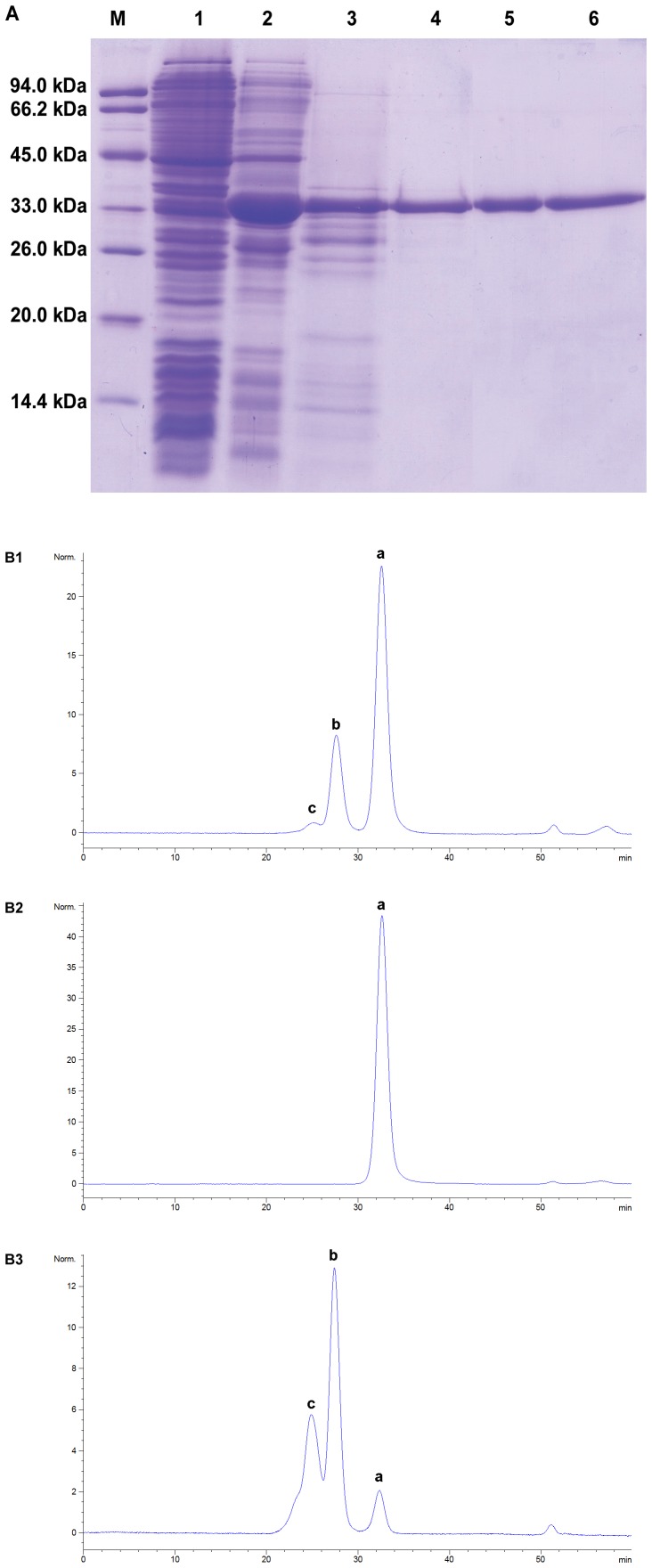
SDS-PAGE and SE-HPLC analyses of rCU during the expression and purification process. (A) SDS-PAGE analysis lanes: M, standard protein molecular weight markers; 1, crude cell extract before IPTG induction; 2, crude cell extract after IPTG induction for 3 hours; 3, crude extraction in 100 mM sodium carbonate buffer (pH 10.3); 4, purified rCU after ammonium sulfate fractionation; 5, purified rCU after xanthine affinity chromatography; 6, purified rCU after anion exchange chromatography. (B) SE-HPLC analysis: B1 purified rCU after xanthine affinity chromatography; B2 tetrameric wDU eluted with 0.1 M NaCl on a Source 15 Q column; B3 large aggregated wDU eluted with 0.25 M NaCl on a Source 15 Q column; a, b and c correspond to tetramer, octamer and large aggregate, respectively.

Commercially activated mPEG always contains a certain amount of PEG diol, ranging between 1 and 10% [16], due to the presence of trace amounts of water during polymerization [17]. PEG diol is difunctional PEG and could induce unwanted cross-linked conjugates during subsequent PEG modification [16]. The amount of cross-linked conjugate dramatically increases as the PEG modification degree increases. In the case of homo-tetrameric uricase of molecular weight 140 kDa, saturated pegylation using all accessible ε amino sites of lysine residues has been developed to reduce its immunogenicity [7], [18]. Moreover, the effective dose of PEGylated uricase [19] is much higher than that of PEGylated cytokines [20]. Taken together, a high modification degree could increase the rate of cross-linked conjugates, while higher dosage means more congregates would be delivered, both of which cause higher immunogenic hazards than conventional PEGylated proteins. To date, most uricase pegylation research has concentrated on the structure and size of the PEG reagent [21], [22] and the improvement of enzymatic and pharmaceutical properties [6], [15]. There are few studies on the potential immunogenic hazards caused by PEG diol-mediated cross-linked conjugates.

**Table 1:** Characteristics of unmodified rCU, PEG reagent and PEGylated rCU proteins.

Items		mPEG-rCU-1	mPEG-rCU-2	mPEG-rCU-3
Unmodified uricase properties	Tetramer content	>99.8%	9.8%	>99.8%
	Enzymatic activity(U/mg)	11.2±0.6	9.3±0.4	11.2±0.6
PEG reagent properties	PEG Diol content	<0.2%	<0.2%	2.7%
PEGylated uricase properties	Modification degree^a^	9.9±0.5	9.7±0.6	10.3±0.6
	Enzymatic activity(U/mg)	9.8±0.7	6.7±0.5	9.6±0.6
	Enzymatic retention	87.5%±5.6%	72.0%±4.6%	85.7%±6.3%

a modification degree: number of 5 kDa mPEG chains that coupled to each uricase monomer.

In this study, canine uricase was selected for recombinant expression and production in *E. coli*. Tetrameric and large aggregated recombinant canine uricase (rCU) proteins were successfully purified and characterized. Prior to PEGylation, anion-exchange chromatography was used to remove the Di-acid PEG (precursor of PEG diol). Uricase with or without aggregates was modified to saturation with purified or unfractionated 5 kDa mPEG-succinimidyl propionic acid (mPEG-SPA). The impact of unmodified uricase aggregates and cross-linked uricase conjugates induced by PEG diol on pharmacokinetics and immunogenicity were studied in vivo. Accelerated blood clearance (ABC) [23], [24], [25], [26], which usually appears with PEGylated nanoparticles but rarely emerges in peg-modified proteins, was confirmed with PEGylated uricase aggregates. The relationship between ABC and the size of PEGylated uricase was investigated.

## Materials and Methods

### Experimental Animals and Ethics Statement

Adult male Sprague-Dawley (SD) rats were obtained from Shanghai B&K Laboratory Animal Co., Ltd. All the animals were kept under a natural dark/light cycle and allowed to access food and water freely. All animal studies were approved by the Animal Care and Use Committee of Fagen Biomedical Inc. (Approval ID: FGB-2009-006) and comply with the USDA Animal Welfare Act <http://www.nal.usda.gov/awic/legislat/usdaleg1.htm> (9 CFR Parts 1, 2, and 3). The administration of substances and removal of blood were in compliance with the EFPIA/ECVAM guidelines issued in 2001 [27].

### Construction, Expression and Purification of Recombinant Canine Uricase (rCU)

A codon-optimized full length canine uricase gene was designed and synthesized based on the protein sequence of wild-type canine uricase [28], [29]. The canine uricase gene was digested and ligated into pET-3c. The recombinant plasmids were transferred to host strain Bl21 Star™ (DE3) plysS. To express the protein, a single colony was inoculated into Luria-Bertani medium containing 0.1 mg/ml ampicillin. When the OD_600_ of the *E. coli* culture reached 0.6, expression was induced by addition of IPTG to the final concentration of 0.4 mM for 5 h at 37°C. The cells were harvested and resuspended in lysis buffers, as described previously [30]. The suspension was centrifuged and insoluble uricase protein was extracted into buffers containing 0.2 M Na_2_CO_3_, pH10.3. A 10% saturated solution of ammonium sulfate was added to the supernatant and the target protein was salted out. The protein was redissolved and loaded onto a xanthine agarose column. Uricase was eluted with an extraction buffer containing 60 µM xanthine. Anion-exchange chromatography (SOURCE 15Q 4.6/100 PE) was then used to separate active uricase isomers. The column was equilibrated with 0.2 M Na_2_CO_3_, pH 10.3. Tetrameric uricase and larger aggregates were eluted with equilibration buffers containing 0.1 and 0.25 M NaCl, respectively.

### Characterization of rCU

The protein concentration of rCU was determined by the Bio-Rad protein assay kit, using bovine serum albumin as the protein standard. The homogeneity and molecular mass of the purified protein were determined by SDS-PAGE. The isomer forms were analyzed by size exclusion HPLC (SE-HPLC) equipped with a UV detector on a Superdex 200 10/300 GL column, which was equilibrated in 0.1 M Na_2_CO_3_, pH 10.3. The sample was eluted at a flow rate of 0.4 ml/min and detected at 280 nm.

The activity of uricase was measured by the decrease in absorbance at 290 nm due to enzymatic oxidation of uric acid, as described previously [31]. Enzyme activity assays were carried out in 0.1 M sodium borate buffer (pH8.6) at 37°C. An extinction coefficient of 12,300 M^−1^ cm^−1^ for uric acid was used. One unit (U) of enzyme activity is defined as the amount of enzyme that catalyzes the oxidation of 1 µmol of uric acid per min at 37°C.

### Preparation of Methoxy-PEG-Succinimidyl Propionate (mPEG-SPA)

Unfractionated dried 5 kDa methoxy-PEG-propionic acid (mPEG-PA) was kindly provided by Jenkem Technology (Beijing, China). The unfractionated mPEG-PA was dissolved in deionized water (5.0 g/L) and purified by DEAE anion-exchange chromatography, which was equilibrated with deionized water. Pure 5 kDa mPEG-PA without Di-acid PEG (PA-PEG-PA, precursor of PEG diol) was eluted with deionzied water containing 10 mM NaCl, and other 5 kDa mPEG-PA that contained Di-acid PEG was eluted with 20 mM NaCl. The Di-acid PEG contents were analyzed by SE-HPLC equipped with a differential refractive index detector on a G2000PW_XL_ column. Purified mPEG-PA that did not contain Di-acid PEG was then precipitated in cold diethylether, filtered and dried over vacuum. Purified and unfractionated mPEG-PA was further converted to mPEG-SPA as described previously [32] and stored at −20°C prior to use.

### Preparation of mPEG-rCU

Purified canine uricase was reacted with mPEG-SPA 5 kDa in sodium carbonate buffer (100 mM, pH 10.0) at 4°C for 12 h. mPEG-SPA 5 kDa (in 4-fold molar excess to the total lysines present in uricase) dissolved in 10 mM HCl was added to a cold solution of rCU (4 mg/ml). The conjugation solutions were loaded onto a Sephacryl S 300 size exclusion column to remove by-products of PEGylation reactions. The column was pre-equilibrated and eluted with PBS. Three types of mPEG-rCU were produced: mPEG-rCU-1 (tetrameric uricase modified with purified mPEG-SPA), mPEG-rCU-2 (aggregated uricase modified with purified mPEG-SPA), and mPEG-rCU-3 (tetrameric uricase modified with unfractionated mPEG-SPA).

### Characterization of mPEG-rCU

The activity of modified uricase was assayed as described above. An Invitrogen NuPAGE™ gradient 4–12% Bis-Tris gel was used to determine the homogeneity of mPEG-rCU. The modification degree (the number of mPEG chain induced in a monomeric uricase protein) was determined by measuring the remaining amino groups on the protein able to react with fluorescamine [33]. The molecular weight of monomeric mPEG-rCU was evaluated by MALDI-TOF. The apparent z-average size (the intensity weighted mean diameter, derived from Stokes-Einstein equation) and polydispersity index (PDI, indicator of conjugate distribution ranging between 0 and 1) of the conjugates were measured by DLS (Malvern Zetasize Nano S90) at a temperature of 25°C and an angle of 90°. In order to verify the result of DSL, the morphology of mPEG-rCU was examined by TEM. The conjugates were mounted on copper grids, negatively stained with 2% phosphotungstic acid and photographed on a Hitachi H-7500 TEM.

**Figure 2:**
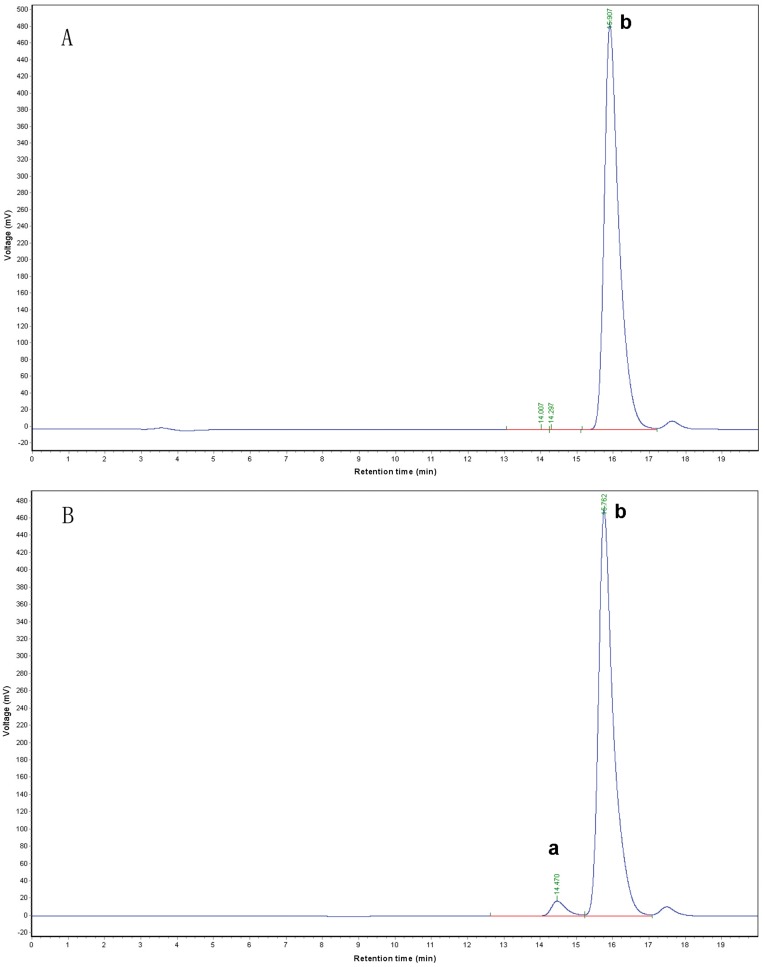
SE-HPLC analysis of purified and unfractionated mPEG-PA. The PEG diol contents were analyzed using a G2000PW_XL_ column with a differential refractive index detector. (A) Purified mPEG-PA; (B) Unfractionated mPEG-PA. a and b correspond to Di-acid PEG and mono-acid mPEG.

**Figure 3:**
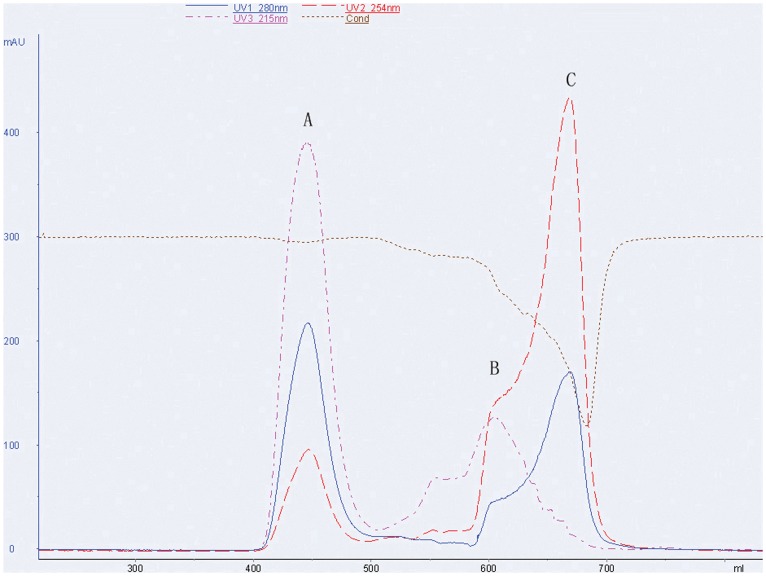
Representative chromatogram of mPEG-rCU purification The three different chromatograms correspond to mPEG-rCU protein (A), unconjugated mPEG (B) and N-hydroxysuccinimide acid (C), respectively.

### Pharmacokinetic Studies

Eighteen male SD rats were randomly divided into three groups and intravenously injected with 1.0 mg/kg of mPEG-rCU-1 (group one) or equal protein amounts of mPEG-rCU-2 (group two) and mPEG-rCU-3 (group three), respectively. Blood samples were taken by retrobulbar bleeding and collected in heparinized vials at 1, 24, 48, 72 and 120 h. The plasma was obtained by centrifugation at 5000 rpm for 10 min. The pharmacokinetics of mPEG-rCU were determined by measuring the amounts of plasma uricase activity as described above. Elimination half life (t_1/2z_) was calculated in a non compartmental manner based on the statistical moment theory by Drug and Statistics for Windows (DAS ver 2.0).

### Immunological Studies

Twenty-four male SD rats were randomly divided into three groups and intravenously injected weekly with 1.0 mg/kg of the three types of mPEG-rCU as described above. Blood samples were collected at 24 h before and after every injection. The serum samples collected before each of four weekly injections were used to determine the antibody amounts. The plasma samples collected after injections were used to determine the pharmaceutical properties.

The amount of IgM and IgG antibodies were measured by enzyme-link immunoassay (ELISA) [34]. Briefly, microtiter plates were treated overnight at 4°C with 250 µl of 50 µg/ml of mPEG-rCU-1, rCU or mPEG-BSA (BSA was modified to saturation with 5 kDa mPEG-SPA and then purified by size-exclusion chromatography) in coating solution (0.1 M NaHCO_3_, pH 9.5). All wells were washed and blocked with 3% BSA in PBS for 2 h at 37°C. Next, the wells were washed and serial dilutions of the serum samples (in PBS) were added to the wells. The plates were incubated for 1 h at 37°C and washed with PBS. Peroxidase conjugated-rabbit anti-rat IgG or IgM diluted 1∶2500 in PBS was added to the wells and the plates were incubated for 1 h at 37°C. The wells were washed and 100 µl of O-phenylenediamine peroxidase substrate solution was added. The enzymatic reaction was stopped after 30 min by addition of 50 µl/well of 2 N sulfuric acid and the absorbance at 490 nm was determined.

**Figure 4:**
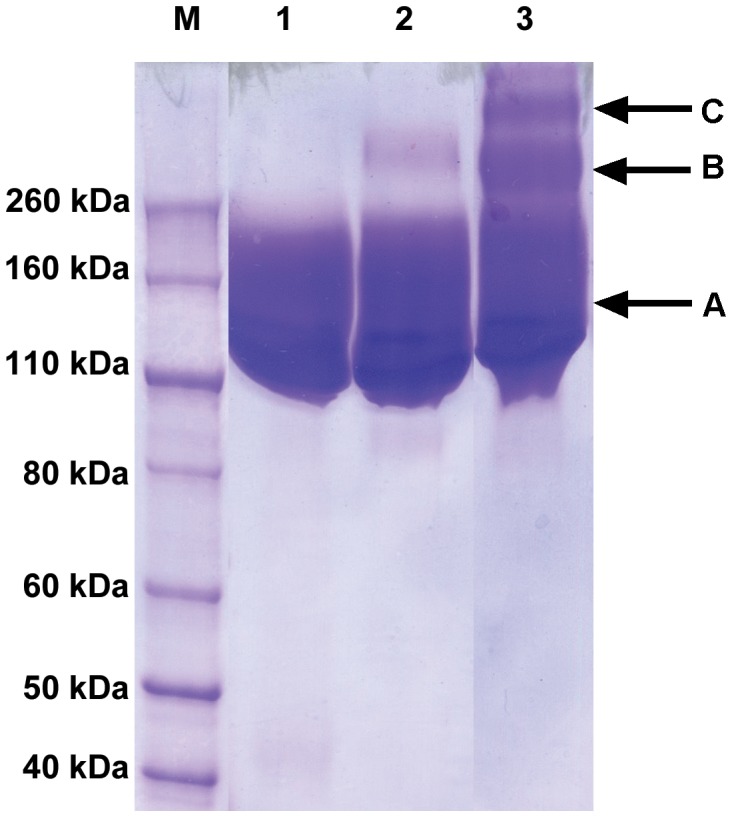
SDS-PAGE analysis of purified mPEG-rCU. Lanes 1, 2 and 3 represent mPEG-rCU-1, mPEG-rCU-2 and mPEG-rCU-3. A, B and C correspond to non-crosslinked PEGylated monomeric rCU, crosslinked conjugates between two PEGylated monomeric rCU, and crosslinked conjugates among three and more PEGylated monomeric rCU, respectively.

To determine if the immune response was neutralizing, the rat anti-serum samples were serially diluted in PBS and incubated with mPEG-rCU-1 for 2 h at 37°C. The enzyme activity was then determined, as described above.

## Results

**Figure 5:**
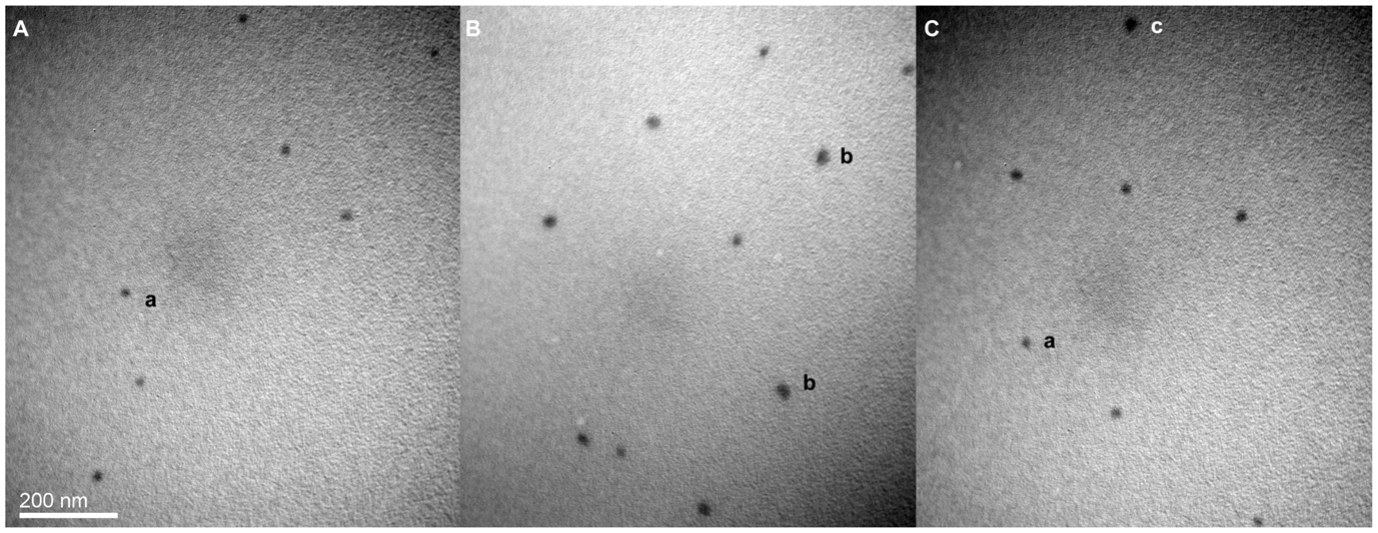
Representative transmission electron micrograph of mPEG-rCU. (A), (B) and (C) represent mPEG-rCU-1, mPEG-rCU-2 and mPEG-rCU-3.The magnification is 100000×, scale bar: 200 nm. a, b and c may correspond to PEGylated tetrameric uricase, PEGylated aggregated uricase, and cross-linked conjugates induced by PEG diol, respectively.

### Expression, Purification and Analysis of rCU

The gene coding for canine uricase was synthesized and fused into pET3c, resulting in the construction of pET3c-rCU. The recombinant enzyme was produced upon induction with IPTG in Bl21 Star (DE3) plysS. As shown in Figure 1A, the amount of synthesized rCU was approximately 35% of the total cellular protein as judged by SDS-PAGE. After ammonium sulfate fractionation, the recovery of total uricase activity was higher than 85% but the recovery of uricase protein (determined by RP-HPLC) was only 34%, suggesting that inactive uricase isomers (such as monomeric uricase) may exist in the initial extractions, but most of them could not be redissolved after ammonium sulfate precipitation. Xanthine agarose was employed to remove the impurities and inactive uricase isomers. The homogeneity of purified rCU was greater than 95% as judged by SDS-PAGE and RP-HPLC [29]. However, SE-HPLC analysis showed that several kinds of active isomers existed (Figure 1B). A Source 15Q column was used to purify the active tetrameric uricase following elution with equilibration buffer containing 0.1 M NaCl. The ratio of the tetramer was greater than 99%, with the above purification method removing most impurities and separating uricase isomers. Enzymatic activity of tetrameric uricase (11.2 U/mg) was higher than that of larger aggregates (9.3 U/mg), eluted with 0.25 M NaCl on the Source 15 Q column, as determined at 37°C, pH 8.6 (Table 1).

**Figure 6:**
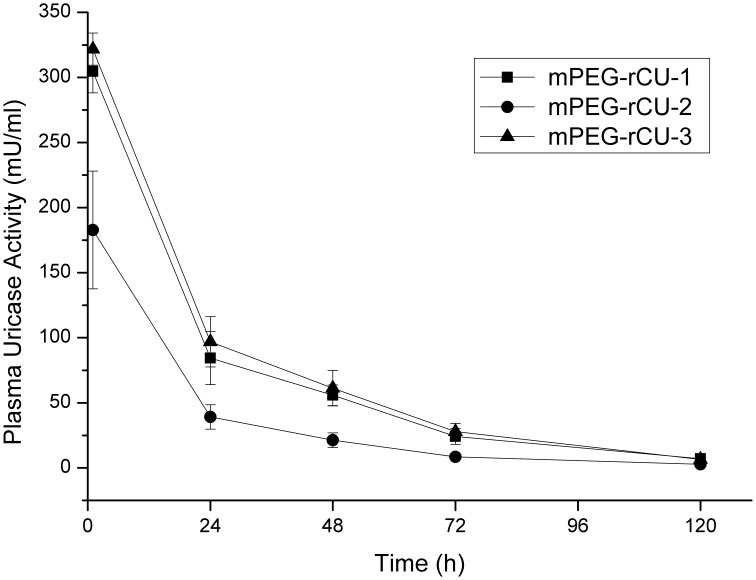
Time courses of plasma uricase activity in three groups after intravenous administration of mPEG-rCU (n = 6).

### Modification and Purification of mPEG-rCU

Based on the differences in their electostatic charge, anion-exchange chromatography was used to remove the Di-acid PEG from the mono-acid mPEG. Di-acid PEG bound more tightly to the anion-exchange column than the mono-acid form. As shown in Figure 2, the content of Di-acid PEG in purified mPEG-PA was lower than 0.2%, as measured by SE-HPLC, whereas the amount in the initial, unfractionated 5 kDa mPEG-PA was about 2.7%. Both purified and unfractionated mPEG-PA were then converted to 5 kDa mPEG-SPA. Following modification, tetrameric and aggregated rCU were separately modified with the purified mPEG-SPA. A further sample of tetrameric rCU was also modified by unfractionated mPEG-SPA, resulting in three types of mPEG-rCU, as shown in Table 1. The three conjugates were further purified by size exclusion chromatography (Figure 3). By-products of PEGylation reactions (e.g. unconjugated PEG and N-hydroxysuccinimide acid) were efficiently removed (for detailed information see material S1). RP-HPLC and SE-HPLC showed that only a single peak existed after the above purification.

### Characterization of mPEG-rCU

**Figure 7:**
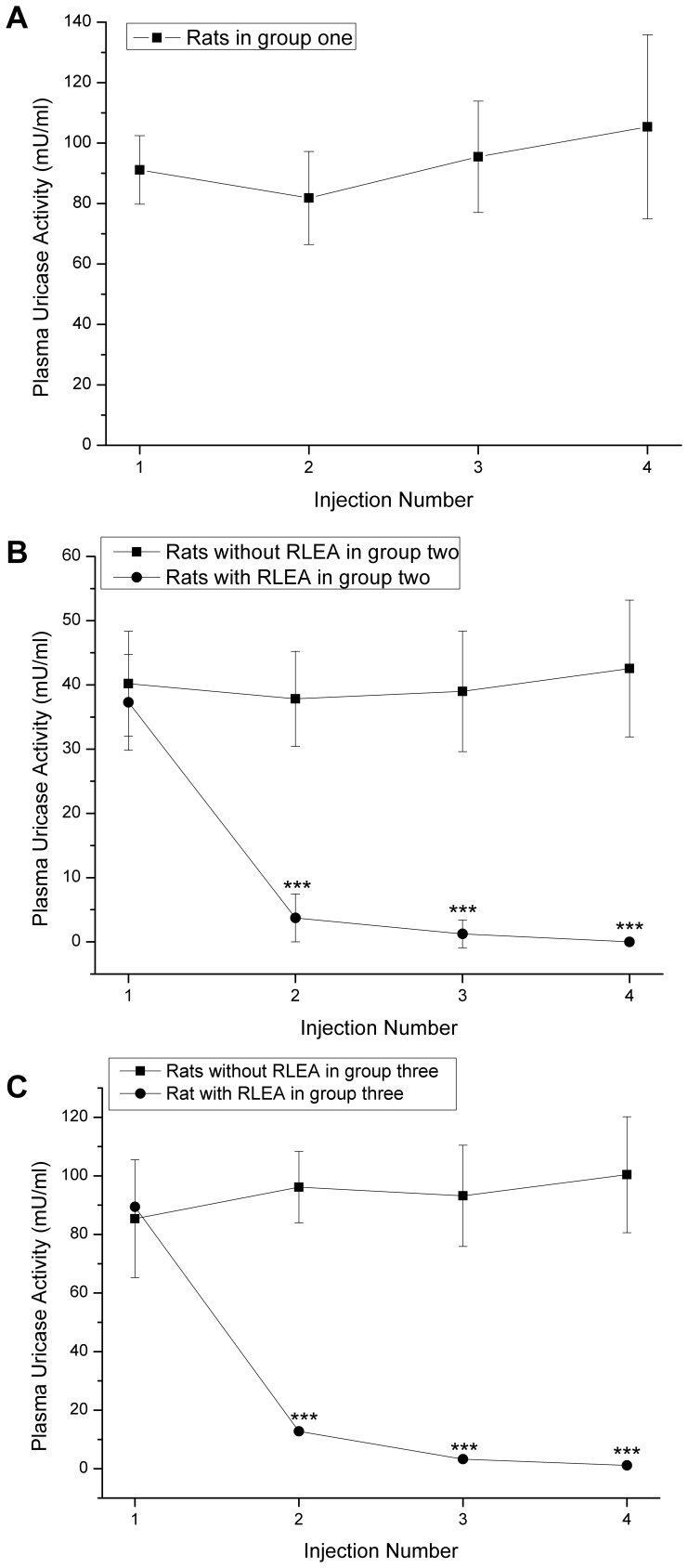
Retention of plasma uricase activity in three groups after different injections of mPEG-rCU. Plasmas were collected 24 hours after each of four weekly injections of mPEG-rCU. *, ** and *** mean significantly different from the same rats before injection at levels of p<0.05, p<0.01, and p<0.001.

A more detailed characterization of 5 kDa mPEG modified canine uricase was presented in a previous study [18]. The results of SDS-PAGE (Figure 4) show that the molecular weight of denatured monomeric mPEG-rCU-1 ranged between 110 and 260 kDa. Such a wide range of molecular weights is higher than the molecular weight of monomeric mPEG-rCU determined by MALDI-TOF (80–90 kDa; for detailed information see material S2) and may be caused by the interference with protein mobility by the coupled mPEG [18]. It should be noted that different SDS-PAGE behaviors among mPEG-rCU-1, mPEG-rCU-2, and mPEG-rCU-3 were observed, which were related to the cross-linked conjugates induced by difunctional PEG diol. No cross-linked conjugates were present in mPEG-rCU-1, resulting in higher protein mobility, whereas cross-linked conjugates were observed in mPEG-rCU-3, as expected, which may have resulted from two or more cross-linked monomers, causing lower protein mobility (for detailed information see material S3). Moreover, the content of cross-linked conjugates in mPEG-rCU-2 was slightly higher than in mPEG-rCU-1, suggesting that unmodified uricase aggregates could increase the cross-linking caused by the low residual amount of PEG diol (<0.2%). The average extents of modification were similar among the three product species (Table 1). About 9–10 5 kDa mPEG chains were coupled to each uricase monomer, suggesting that there was little impact of uricase aggregation and PEG diol on modification characteristics. The enzymatic retentions of mPEG-rCU-1 and mPEG-rCU-3 were higher than 85.0%, whereas that of mPEG-rCU-2 was only 72.0%, indicating that unmodified uricase aggregates could affect enzymatic retention during PEGylation.

**Figure 8:**
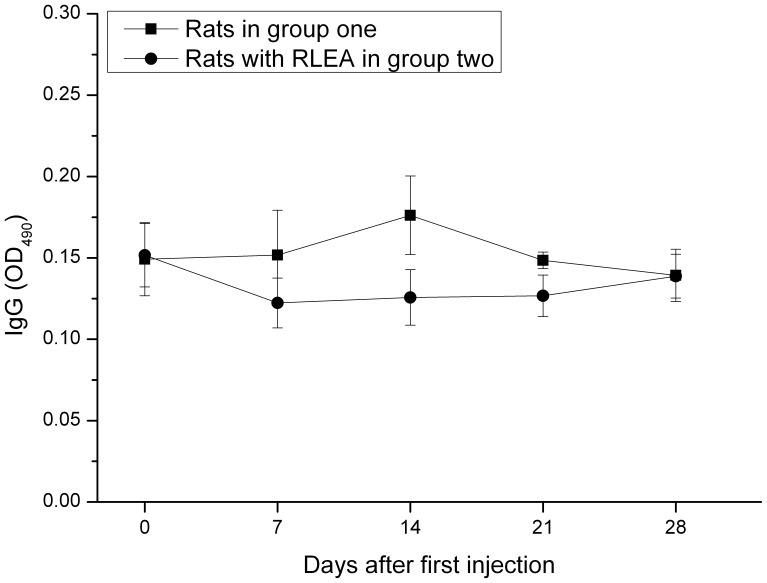
ELISA analysis of IgG antibody against mPEG-rCU. Serum samples were collected 24 hours before each of four weekly injections of mPEG-rCU. Microtiter plates were coated with 50 µg/ml of mPEG-rCU-1.

**Figure 9:**
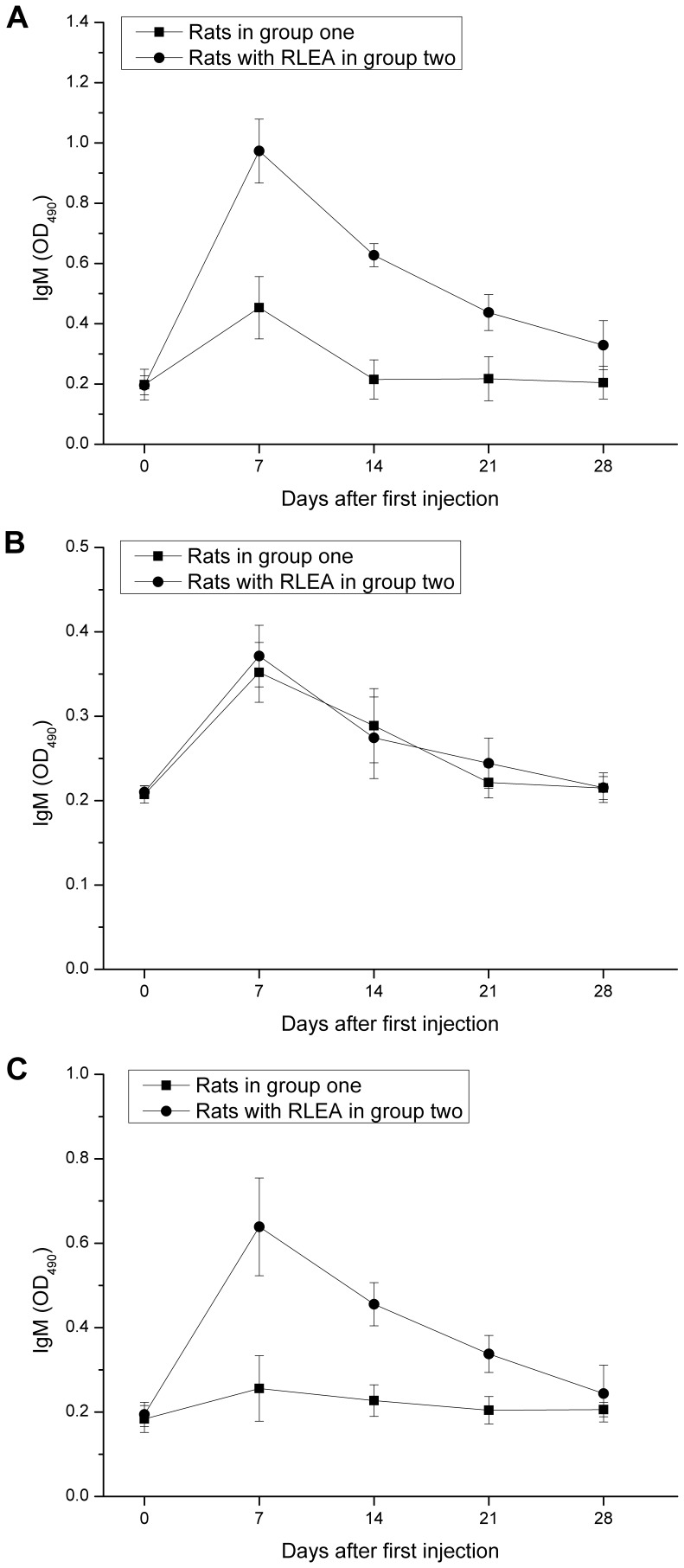
ELISA analysis of IgM antibody against mPEG-rCU, rCU, or mPEG-BSA Serum samples were collected 24 hours before each of four weekly injections of mPEG-rCU. (A) Microtiter plates were coated with 50 µg/ml of mPEG-rCU-1. (B) Microtiter plates were coated with 10 µg/ml of tetrameric rCU. (C) Microtiter plates were coated with 50 µg/ml of mPEG-BSA.

The size distribution of the three conjugates was measured by DLS. Only one peak was observed in all three mPEG-rCUs because of the lack of baseline resolution (data not shown) [35] so it is difficult to distinguish different populations. The main differences were the apparent z-average size and PDI values. The z-average sizes of mPEG-rCU-1, mPEG-rCU-2, mPEG-rCU-3 were 17.68, 38.56, and 22.38 nm, respectively. The PDI of mPEG-rCU-1 was 0.062, confirming the good particle uniformity of 5 kDa mPEG modified tetrameric uricase, whereas the PDI of mPEG-rCU-2 and mPEG-rCU-3 were 0.519 and 0.287, indicating that a broad size distribution could be induced by large aggregated uricase and cross-linked uricase conjugates. To compare the morphological diversity, each conjugate was separately examined by TEM. As shown in Figure 5, homogeneous particles with diameters of about 14–18 nm (indicated by ‘a’ in Figure 5A) were observed in mPEG-rCU-1, which may correspond to 5 kDa mPEG modified tetrameric uricase. Many larger particles with diameters from 30 to 50 nm (indicated by ‘b’ in Figure 5B), which may correspond to aggregated conjugates, were observed in mPEG-rCU-2, whereas only a small number of large particles were observed in mPEG-rCU-3 and most of the particle size was essentially the same as that in mPEG-rCU-1.

### Pharmacokinetic Studies

Rats were used as an experimental model to evaluate the pharmacokinetics of mPEG-rCU. Note that the known effective human dose for PEGylated uricase is about 1.0–2.0 U/kg [18], [19]. We estimated 0.2 mg/kg (2.0 U/kg) as the effective dose of mPEG-rCU for individual humans. Therefore, a dose of 1.0 mg/kg was used for the rats, which were intravenously injected with the three different mPEG-rCUs, as described above. The time course profiles obtained by enzymatic activity determination in plasma are depicted in Figure 6. The elimination half-lives for mPEG-rCU-1, mPEG-rCU-2, mPEG-rCU-3 were 26.8, 24.8 and 20.1 h, respectively, indicating that large aggregated uricases and cross-linked conjugates induced by PEG diol did not dramatically affect the circulation half-life after the first injection.

Repeated injection experiments showed that there was no change in multiple pharmacokinetic properties of rats in group one (administered with mPEG-rCU-1) during the next three repeat injections (Figure 7A). In contrast, the circulatory enzymatic activities of five rats (Figure 7B) in group two (administered with mPEG-rCU-2) were lost rapidly after the second injection and the disappearance rates of enzymatic activities increased with the next repeated injection. Only one rat in group three (administered with mPEG-rCU-3) showed rapid loss of enzymatic activity (RLEA) after the second injection (Figure 7C). No new rats in groups two and three displayed rapid loss of enzymatic activity during later injections.

mPEG-rCU-1 was injected into the rats of group two, which had displayed RLEA prior to this injection. A similar RLEA phenomenon was also observed (data not shown), indicating that this effect, which may be triggered by aggregated mPEG-rCU, could also influence the circulation of normal tetrameric mPEG-rCU.

### Immunological Studies

No difference in enzymatic activity was observed after incubating mPEG-rCU with anti-serum obtained from all three administration groups and PBS, suggesting that no neutralizing antibodies were found in any of the rats during the four injections of mPEG-rCUs and that the RLEA was not related to neutralizing antibodies.

The serum levels of IgG and IgM antibodies reactive to mPEG-rCU after every repeat injection were measured by ELISA. A screening ELISA performed on 1∶50 and 1∶100 dilutions failed to detect IgM and IgG antibodies against mPEG-rCU-1. The next screening was performed at 1∶10 dilutions of plasma. As shown in Figure 8, no obvious IgG antibodies were observed in either the normal rats or those that displayed RLEA during the four injections, suggesting that the RLEA was not mediated by anti-mPEG-rCU IgG antibodies. However, there were significant differences in the amounts of anti-mPEG-rCU IgM antibodies measured between the normal rats in group one and the rats in group two, which displayed RLEA (Figure 9A). The amount of IgM antibody reactive to mPEG-rCU in all cases reached its maximum level before the second injection (at day 7) and decreased in later injections. Only the rats in group two that displayed RLEA gave a positive response during the injections (where anti-mPEG-rCU IgM was regarded as positive if the optical density at 490 nm wavelength (OD_490_) was more than 2.1 times that of the same rats before injection). The amounts of IgM antibody in group one rats showed a slight increase but they nevertheless showed a negative response after the first injection. Similarly positive IgM antibodies were observed in one rat of group three, which also showed RLEA (data not shown).

Another experiment was carried out to specify the anti-mPEG-rCU antibodies. The amounts of IgM antibody against rCU proteins and mPEG were evaluated by using rCU and mPEG-BSA, respectively, for ELISA microplate coating. As shown in Figure 9B, no positive IgM antibodies were observed by coating rCU and there were no significant differences in OD_490_ between the rats with and without RLEA, indicating that the protein portion of mPEG-rCU did not react with the anti-mPEG-rCU IgM antibodies. In contrast, positive IgM antibodies were observed by coating mPEG-BSA for rats that showed RLEA (Figure 9C), whereas the rats without RLEA still displayed a negative response, suggesting that the positive IgM antibody observed in group two was against mPEG portion of mPEG-rCU.

## Discussion

Recombinant canine uricase was expressed and purified to homogeneity. Several forms of rCU existed in the extraction buffers. The monomers were inactive forms that could be easily separated by ammonium sulfate fractionation and affinity chromatography. The octameric uricase and larger aggregates were active isomers, which could only be detected by SE-HPLC. In this study, tetrameric and larger aggregated rCU were successfully evaluated and separated.

PEGylation has been successfully used to enhance the therapeutic potential of many proteins. The molecular mass of unmodified uricase is about 140 kDa, while proteins whose molecular mass exceeds 100 kDa are efficient inducers of immune responses [36]. The larger the PEG used in modification, the more the opportunities that may arise to trigger immune responses. The currently marketed PEGylated uricase (Pegloticase) is modified with 10 kDa mPEG and the average molecular weight is approximately 540 kDa [9], which may explain why 92% of patients developed antibodies against it [9]. Moreover, because the antibodies were mainly against the PEG moiety of Pegloticase, using 5 kDa mPEG may reduce the generation of anti-PEG antibodies and lower the overall immunogenicity of PEGylated uricase. Therefore, 5 kDa mPEG, which has been used in the manufacture of several commercial PEGylated proteins [37], [38], [39], was selected for further investigation in this research. Low enzymatic retention was the largest obstacle in developing a 5 kDa PEG modified uricase [22], [40]. In this study, the enzymatic retention of PEGylated tetrameric rCU was higher than 85.0%, whereas that of PEGylated rCU aggregates was only 72.0%, suggesting that large isomers could affect enzymatic retention but the impact could be resolved after removing the aggregates.

Commercially available mPEG contains a considerable amount of PEG diol, which may yield unwanted cross-linked conjugates. The amount of cross-linked isomers induced by PEG diol could increase dramatically with an increasing degree of modification. The theoretical maximum amounts of cross-linked conjugates may be calculated by the following equation:

C = 2×the number of PEGs coupled per protein×PEG diol content.

Supposing that the amount of PEG diol is 2%, when a protein is coupled with only one polymer chain, the maximum content of cross-linked protein may reach 4%; whereas, in the case of highly PEGylated uricase, where one monomeric uricase is coupled with 9–10 5 kDa mPEGs, the theoretical maximum amount of cross-linked protein may reach 40%. Removing PEG diol is important for developing homogeneous mPEG-rCU. mPEG-SPA is unstable in water and there is no mechanism for removing PEG diol from the activated mPEG reagents [41]. On the contrary, mPEG-PA is a stable intermediate formed during the activation step. Anion-exchange chromatography was successfully used to remove Di-acid PEG-PA from the mono-acid mPEG-PA in this study.

The improvement of the optimized PEGylated uricase and the impact of uricase aggregates and cross-linked conjugates on pharmaceutical and immunological properties were investigated using rats. Rapid loss of enzymatic activity was observed in rats injected with mPEG-rCU-2 and mPEG-rCU-3 after the second injection, indicating that both uricase aggregates and cross-linked conjugates could trigger immune responses and influence pharmacokinetics. Moreover, the impact of large aggregated uricase was larger than cross-linked conjugates, as indicated by the different occurrence rates of such phenomena between rats administered with mPEG-rCU-2 and mPEG-rCU-3. No neutralizing antibodies were detected in the animals, indicating that the RLEA is caused by accelerated clearance from circulation. The difference in serum levels of IgM antibodies between the rats in group one and the rats displaying rapid elimination in group two indicates that IgM antibodies are responsible for the RLEA after repeated injections. Moreover, based on the specificity of anti-conjugate IgM antibodies, we speculated that the RLEA is induced by anti-PEG IgM antibodies.

The accelerated blood clearance phenomenon has previously been observed upon repeated injection of PEGylated liposomes [25], [26], [42], [43]. The apparent time of the RLEA and the changes in properties of the IgM antibodies observed here were consistent with those observed in PEGylated liposomes. Thus, we speculated that the mechanism causing the RLEA is the same as that causing ABC. However, previous studies have shown that the mechanism(s) for inducing such behavior are still not entirely clear. The hypothesis proposed is that this behavior involves sequential steps, including induction of anti-PEG IgM antibody production in the spleen with the first dose of PEGylated conjugates, complement activation by the IgM antibody and opsonization by C3 fragments following the second dose of PEGylated conjugates, and their uptake by the mononuclear phagocyte system [24], [44], [45], [46].

The ABC phenomenon is generally induced by PEG-modified nanoparticles but a similar phenomenon was first confirmed in PEGylated proteins. Earlier reports demonstrated that various factors, such as dose, size and peg-surface density, could influence the magnitude of the ABC [23] but strategies to avoid it remains unclear. The shared properties between PEGylated liposomes and uricase include the large size of the conjugates. The z-average size of 5 kDa mPEG-modified tetrameric uricase calculated by DLS (17.68 nm) agreed essentially with that evaluated by TEM (14–18 nm). Although the z-average size of mPEG-rCU-2 was only 38.56, the size of large aggregates in mPEG-rCU-2 caused by aggregation of several tetrameric uricase constructs may increase several times and reach 60 nm, which is consistent with the size of small PEGylated liposomes [47] that can effectively manifest such behavior. On the contrary, PEGylated tetramer conjugates apparently could not induce ABC behavior (rats in group one). In addition, most of the cross-linked conjugates induced by PEG diol were aggregates between two tetramers, whose size may reach 30–40 nm but are less efficient in triggering that behavior (rats in group three). The above observations indicate that the conjugate size is of primary importance in inducing the ABC phenomenon, which is consistent with the results reported by Koide et al [48], [49]. To be more precise, we speculate that 40–60 nm is the lower size limit that can trigger ABC. PEGylation cannot induce ABC when the size of the PEGylated protein is under this lower limit and this may explain why ABC is not widely found in PEGylated proteins. However, for uricase, native aggregates and cross-linked conjugates may attain the lower size limit and high usage may then ensure the dose is sufficient to induce ABC.

Although the uricase origin (canine) used in this study differs from that of Pegloticase (porcine), the amino acid identity between porcine and canine uricase is higher than 90%. Moreover, the immunogenicity of native uricase could be sufficiently reduced by PEGylation. Thus, it is likely that uricases from different mammalian origin differ little in their influence on immunogenicity. The Stokes’ radius of native tetrameric uricase is 42.7 Å [21], while the particle size reached 14–18 nm after modification with 5 kDa mPEG, which is approximately 2–3 times larger than the native form and is in good agreement with the results reported by Efremova et al. that modification with 5–6 kDa mPEG could create a polymer layer thickness of 4–10 nm [50]. The size may increase 4–10 times by modification with 10 kDa or 20 kDa mPEG, and may thus reach the lower size limit that can trigger ABC. Since the a similar decreased urate-lowering efficacy after repeated administrations observed during clinical trials of Pegloticase, which is modified by 9–10 10 kDa mPEG chains per monomer, we hope that our findings in rats may help to explain the decreased urate-lowering efficacy of Pegloticase in clinical practice. However, further investigation is required to provide direct evidence.

It should also be noted that ABC, triggered by larger aggregates, could also influence the circulation of the smaller PEGylated uricase, suggesting that if we wish to avoid such behavior, larger sized conjugates should be sufficiently removed. Previous studies ignored this behavior and this may have prevented further development of PEGylated uricase for clinical use. In this study, we used anion exchange chromatography to remove uricase aggregates and to purify 5 kDa mPEG-SPA. After removing the uricase aggregates and the PEG diol contaminant and modifying with small PEG reagents, the size of the mPEG-rCU remained below the lower limit that can trigger ABC, thus successfully avoiding ABC. Furthermore, the optimized conjugates stimulated few IgM and IgG antibodies after four injections, indicating the effective reduction of immunogenicity. Further chronic toxicity and immunogenicity assessments in rodents and non-human primates are currently underway in our laboratory, with the ultimate goal of bringing the PEGylated uricase to a successful clinical application.

## Supporting Information

Material S1 **Homogeneity analysis of purified mPEG-rCU.**
(DOC)

Material S2 **MALDI-TOF analysis of mPEG-rCU.**
(DOC)

Material S3 **SDS-PAGE analysis of mPEG-rCU with different contents of PEG diol.**
(DOC)
