# Supplementary material for: Impact of Large Aggregated Uricases and PEG Diol on Accelerated Blood Clearance of PEGylated Canine Uricase
Source: PLoS One. 2012 Jun 26;7(6):e39659. doi: 10.1371/journal.pone.0039659 (PMC3383732; doi:10.1371/journal.pone.0039659)
Supplement: Material S1 — Homogeneity analysis of purified mPEG-rCU. (DOC) [file pone.0039659.s001.doc]

**Homogeneity analysis of purified mPEG-rCU**

Non-conjugated mPEG-SPA undergoes rapid hydrolysis in water. Its main hydrolysis products are inactivated PEG and N-hydroxysuccinimide acid , which are also the main by-products of PEGylation reactions. Because of the large hydrodynamic radii of PEGylated uricase, size-exclusion chromatography (SEC) was used to remove the free PEG and N-hydroxysuccinimide acid. Free PEG does not have UV absorption (Figure 1B) but could be analyzed by Evaporative Light Scattering Detector (ELSD) (Figure 1A), while N-hydroxysuccinimide acid is a small five-membered ring compounds and could be easily analyzed by UV detector. Sephacryl S 300 size exclusion column was used to remove by-products of PEGylation reactions, and reverse-phase HPLC (RP-HPLC) with ELSD and UV detectors were used to monitor the efficiency of SEC. As shown in Figure 2, unpurified mPEG-rCU reaction solutions contains lots of N-hydroxysuccinimide acid (Fingure 2B, peak a) and free PEG (Figure 2A, peak b). After purification by SEC, no N-hydroxysuccinimide acid could be detected by UV detector (Figure 3B). SE-HPLC was also used to detect the remnant N-hydroxysuccinimide acid, none small compound absorption could be detected at 214 and 280 nm (Figure 4), indicating that N-hydroxysuccinimide acid was effectively removed by SEC. Only slightly amount of free PEG could be detected by ELSD (Figure 3A, peak b) , and the remaining content of PEG is lower than 0.1 mg/ml (Figure 5, the remaining peak area of PEG in Figure 3A is smaller than that in Figure 5), while the initial concentration of free PEG in reaction solutions is higher than 60 mg/ml, indicating that most of free PEG (>99%) was removed by SEC.

Analytical RP-HPLC was performed using a Waters Symmetry 300TM C_4_ analytical column (4.6 x 150 mm, 5 µm) with a 30-min 5-95% linear gradient of acetonitrile, 0.1% trifluoroacetric acid, at a flow rate of 1 ml/min. Elution was monitored by Waters 2487 dual-wavelength absorbance detector (214 nm) and Varian 380-LC ELSD.

Analytical SE-HPLC was performed on a Superose-6 10/300 GL column(Amersham Biosciences) using PBS, pH7.4 as mobile phase. The sample was eluted at a flow rate of 0.4 ml/min and detected at 214 and 280 nm using an Agilent 1100 system.


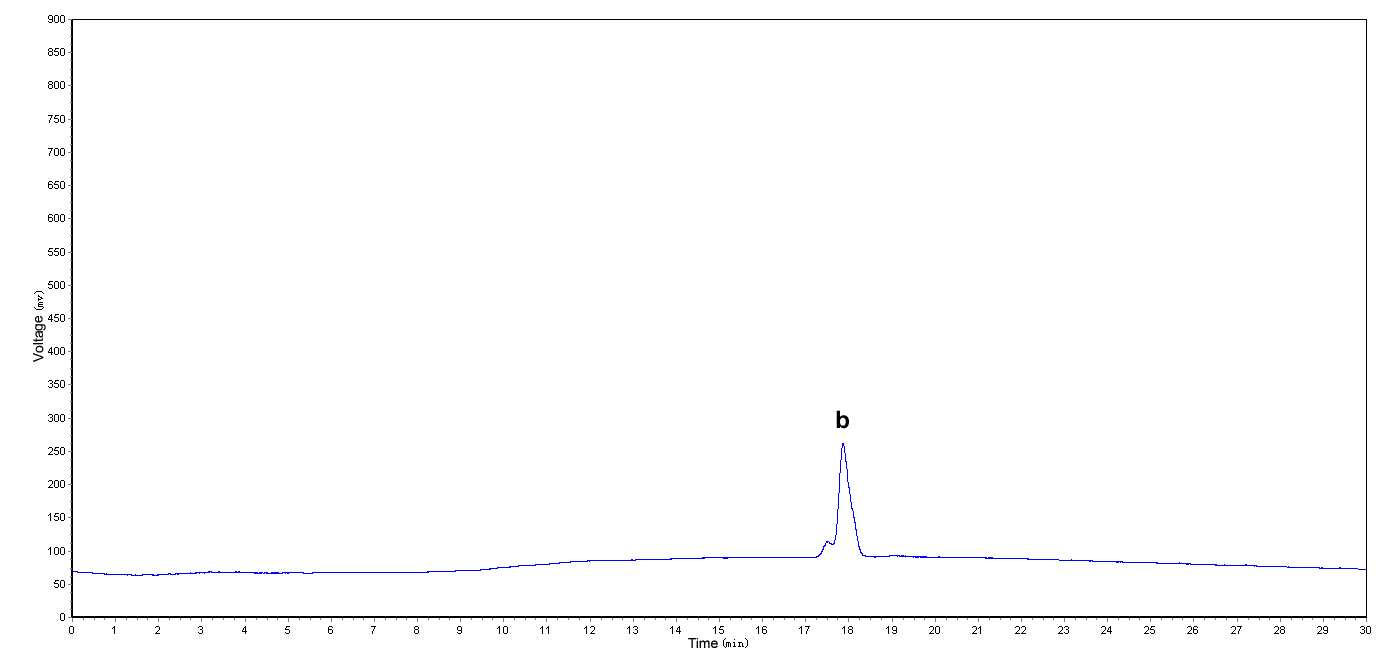


**1A:ELSD**


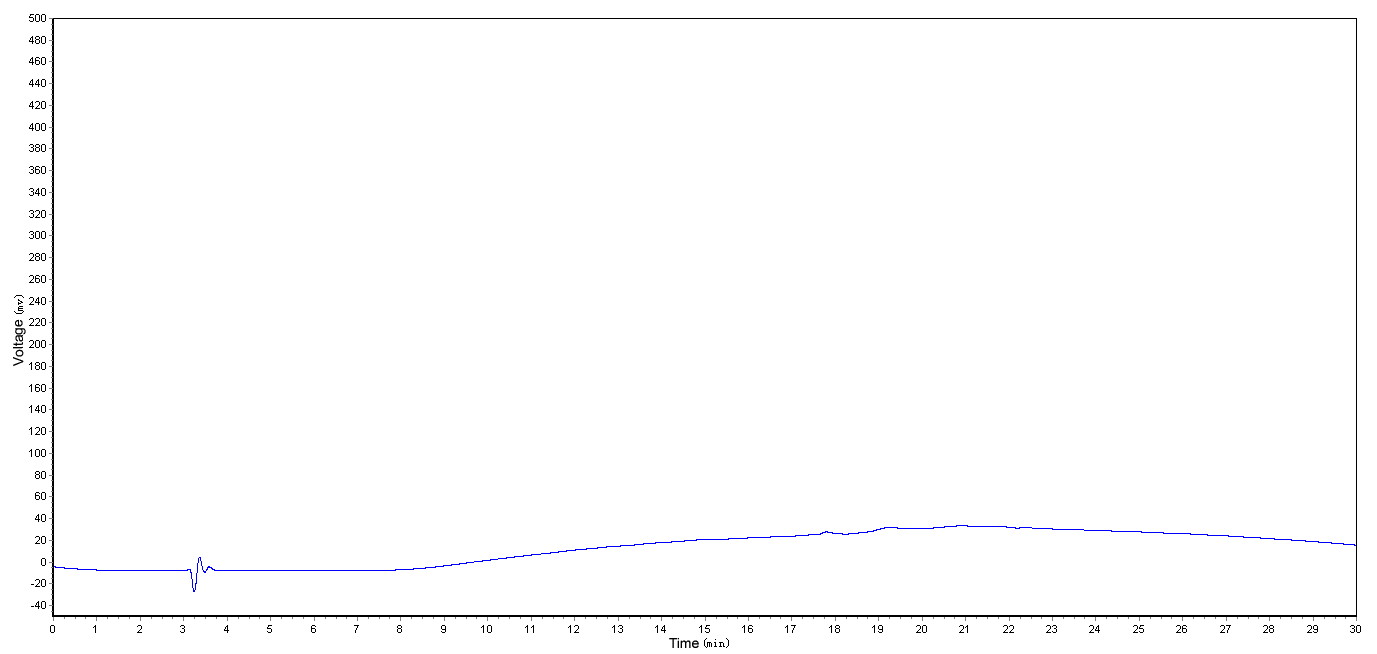


**1B:UV**

Figure 1 RP-HPLC analysis of mPEG-SPA (1mg/ml)

b corresponds to 5 kDa mPEG-SPA


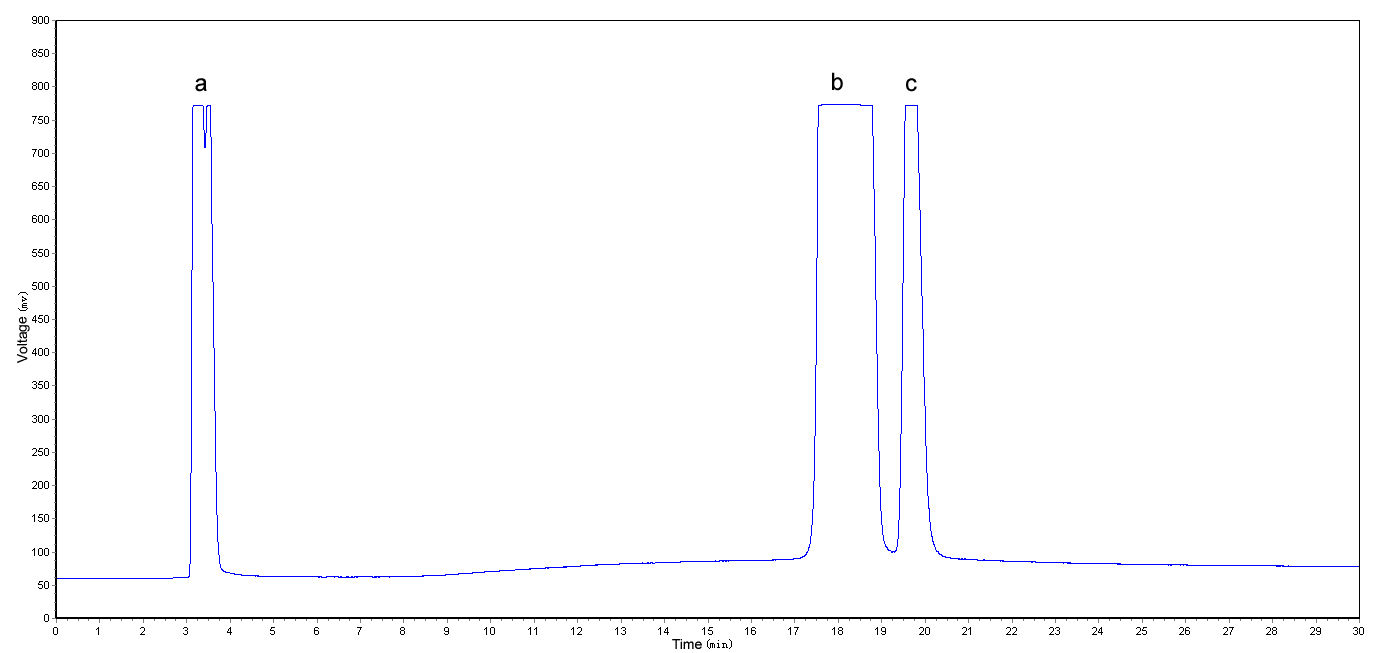


**2A:ELSD**


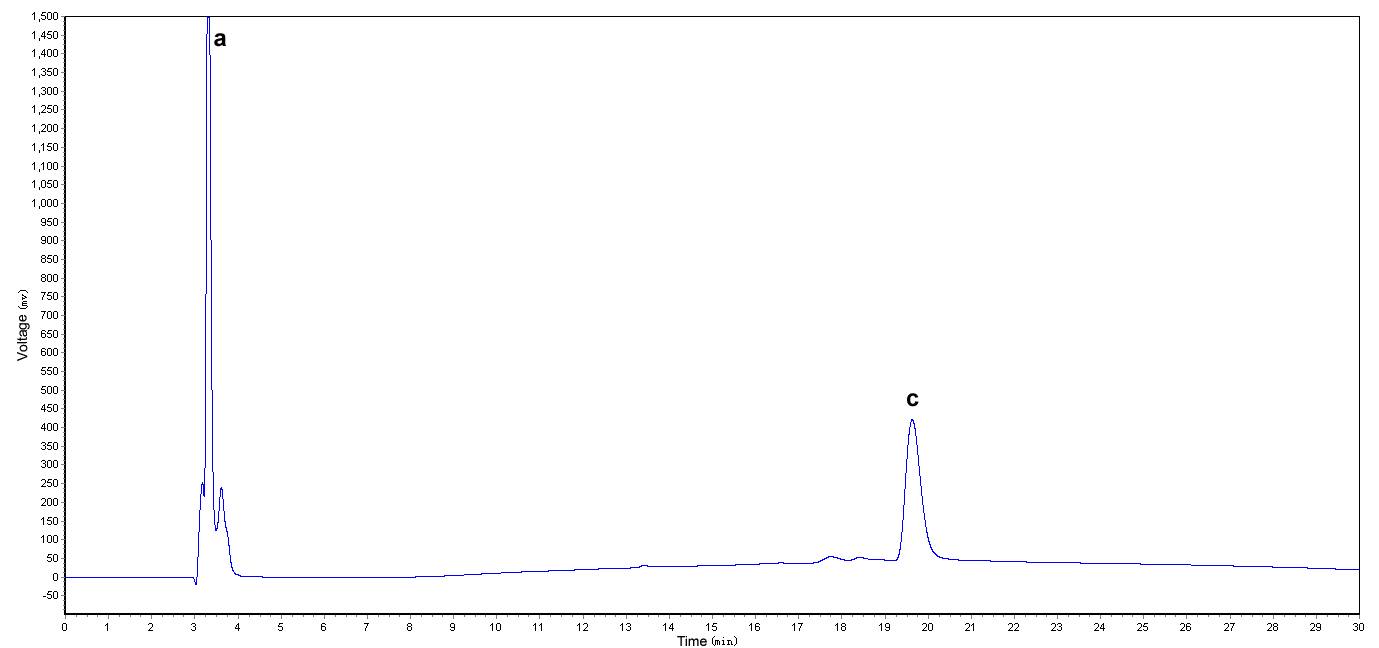


**2B:UV**

Figure 2 RP-HPLC analysis of modified mPEG-rCU without purification(1mg/ml) a, b and c correspond to N-hydroxysuccinimide acid, free peg and mPEG-rCU proteins, respectively.


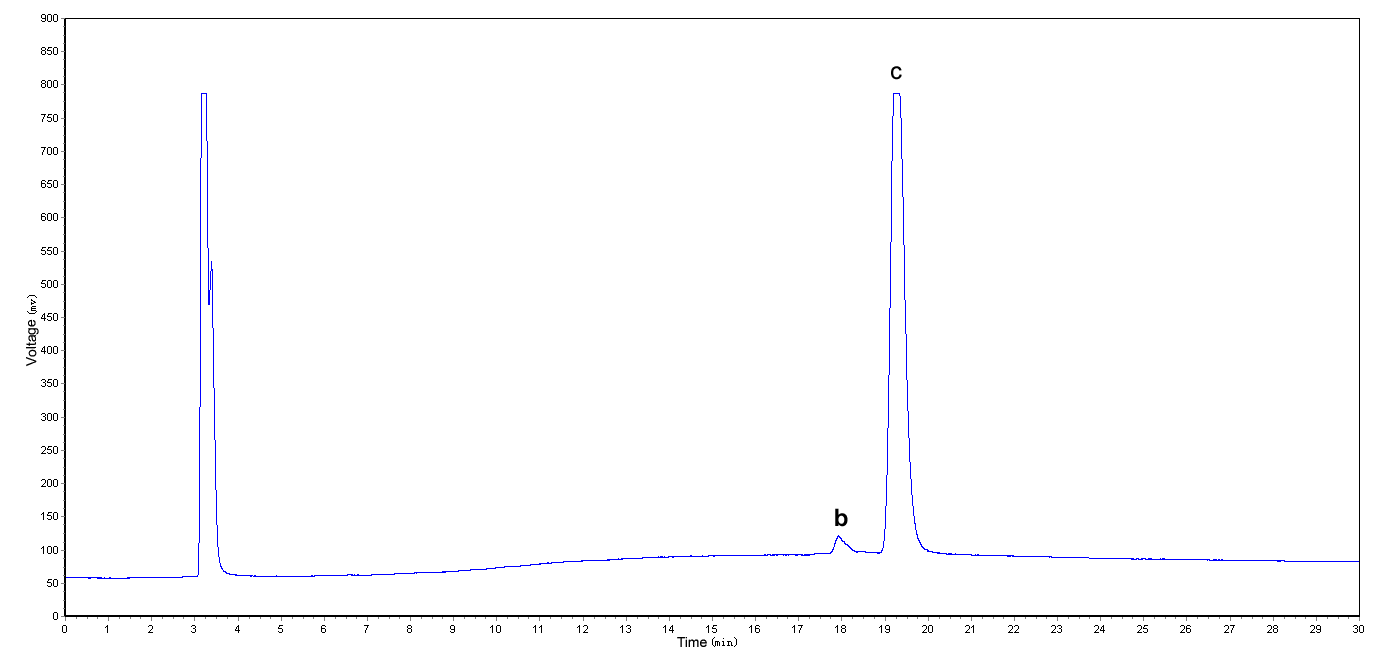


**3A:ELSD**


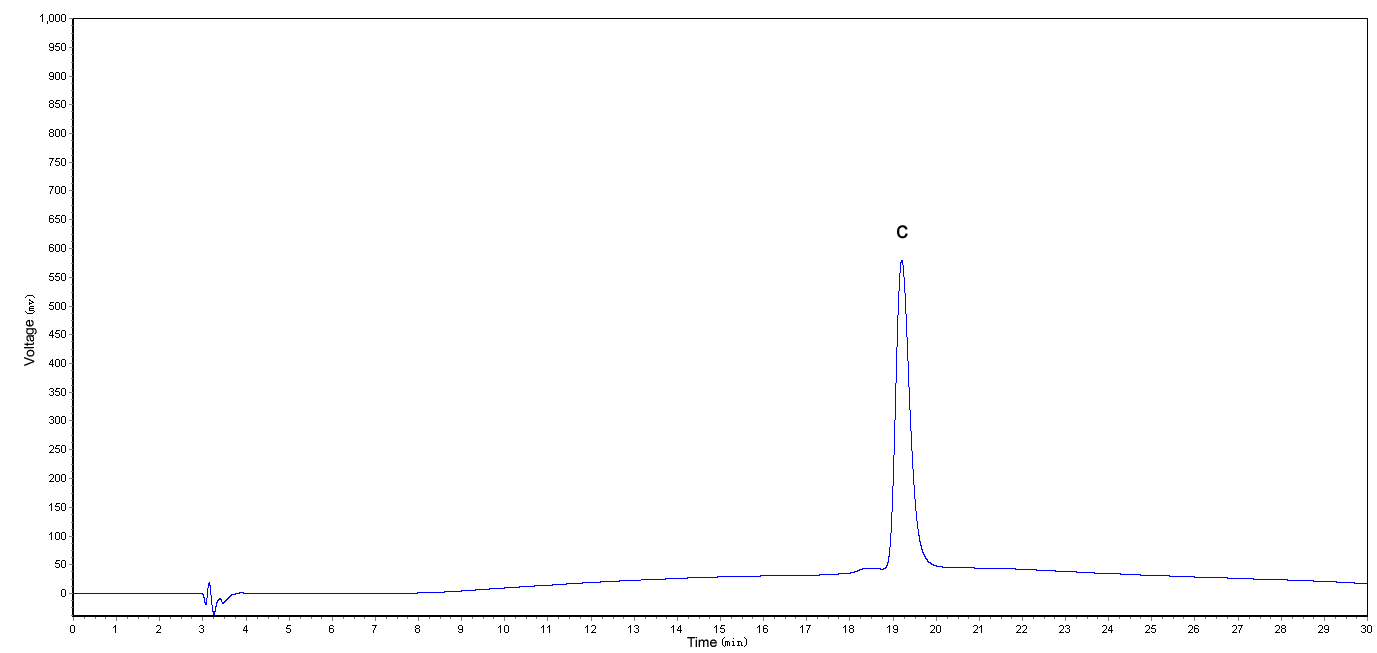


**3B:UV**

Figure 3 RP-HPLC analysis of purified mPEG-rCU (1mg/ml)

b and c correspond to free peg and mPEG-rCU proteins.

Figure 4 SC-HPLC analysis of purified mPEG-rCU (4mg/ml)


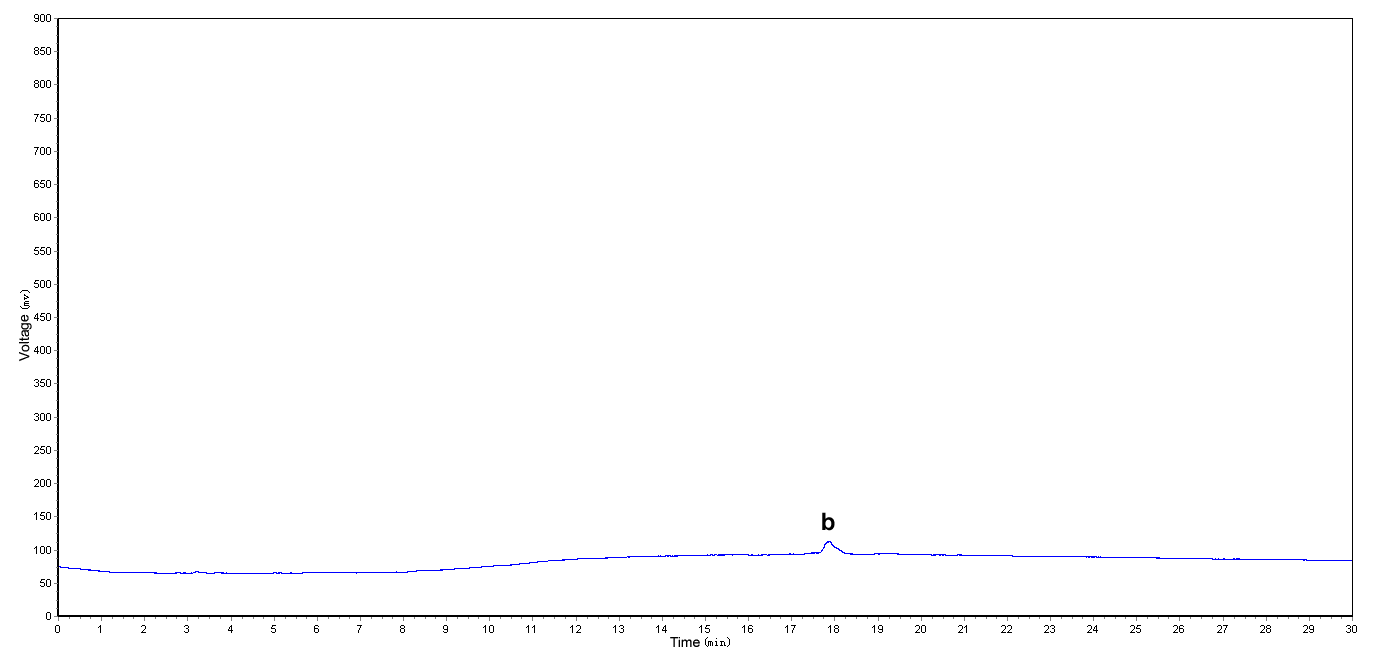


**ELSD**

Figure 5 RP-HPLC analysis of mPEG-SPA (0.1mg/ml)

b corresponds to 5 kDa mPEG-SPA

(1) Fee, C. J., and Van Alstine, J. M. (2004) Prediction of the viscosity radius and the size exclusion chromatography behavior of PEGylated proteins. *Bioconjug Chem* *15*, 1304-13.
