# Supplementary material for: Impact of Large Aggregated Uricases and PEG Diol on Accelerated Blood Clearance of PEGylated Canine Uricase
Source: PLoS One. 2012 Jun 26;7(6):e39659. doi: 10.1371/journal.pone.0039659 (PMC3383732; doi:10.1371/journal.pone.0039659)
Supplement: Material S2 — MALDI-TOF analysis of mPEG-rCU. (DOC) [file pone.0039659.s002.doc]

**MALDI-TOF analysis of mPEG-rCU**

Mass spectrometry was performed on a Bruker AutoflexⅡ MALDI–TOF mass spectrometer, which utilized a solid-state laser (Nd:YAG, 355 nm), used sinapinic acid (SA) as matrix, and operated in linear, positive ion mode with a static accelerating voltage of 20 kV. As shown in Fig. 1, the molecular weight of mPEG-rCU-1 evaluated by MALDI-TOF is about 86061 Da, which coincide well with the molecular weight calculated from the average modification degree that tested by fluorescamine: 35 kDa (protein Molecular weight)+10(the number of mPEG chain induced in a monomeric uricase protein)×5 kDa ( PEG Molecular weight)= 85 kDa.


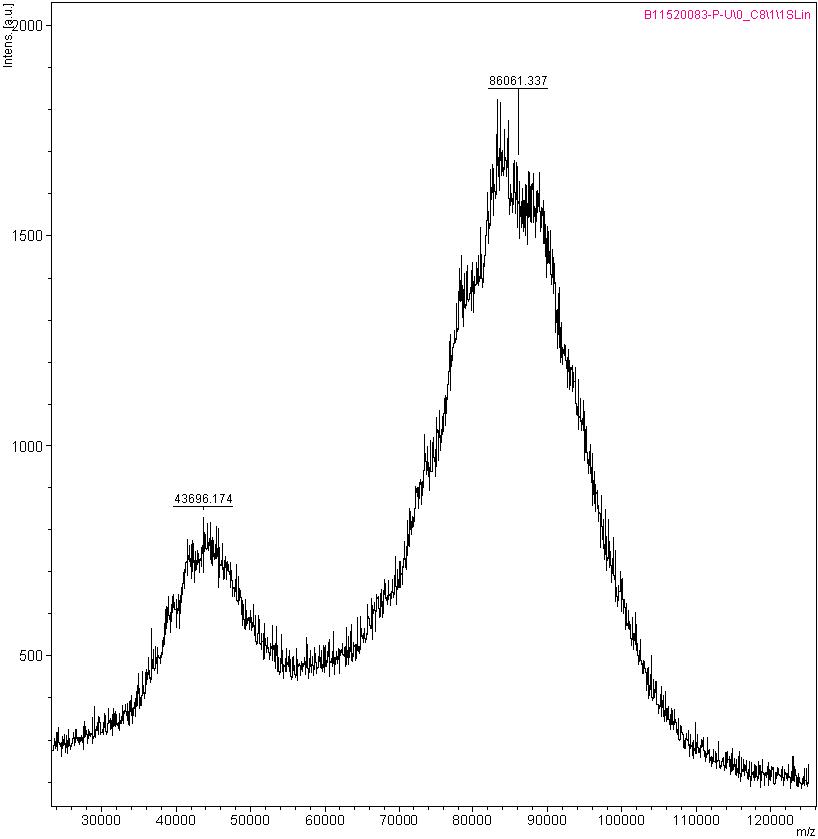


**Fig.1 MALDI-TOF mass spectra of mPEG-rCU-1**
