# Supplementary material for: Impact of Large Aggregated Uricases and PEG Diol on Accelerated Blood Clearance of PEGylated Canine Uricase
Source: PLoS One. 2012 Jun 26;7(6):e39659. doi: 10.1371/journal.pone.0039659 (PMC3383732; doi:10.1371/journal.pone.0039659)
Supplement: Material S3 — SDS-PAGE analysis of mPEG-rCU with different contents of PEG diol. (DOC) [file pone.0039659.s003.doc]

**SDS-PAGE analysis of mPEG-rCU with different contents of PEG diol**

In order to clarify the different PAGE behaviors among different mPEG-rCUs, 5 kDa mPEG-SPA with different amounts of PEG diol were obtained by mixing the purified mPEG-SPA with unfractionated mPEG-SPA containing about 2.7% of PEG diol. Purified tetrameric rCU proteins were modified with the above mPEG-SPA, respectively. As shown in below figure, the higher bond (indicated as B) was observed (Lanes3-5) and its amount increased as the PEG diol content increases. We speculated that such bond was cross-linked by two monomeric rCU proteins. New higher bond ( indicated as C ) were observed (Lanes 6 and 7) with further increase of the concentration of PEG diol. We speculated that such bond was cross-linked among three PEGylated monomeric rCU proteins. Moreover, some higher bonds still emerged in lanes 6 and 7, which may correspond to larger cross-linked conjugates.


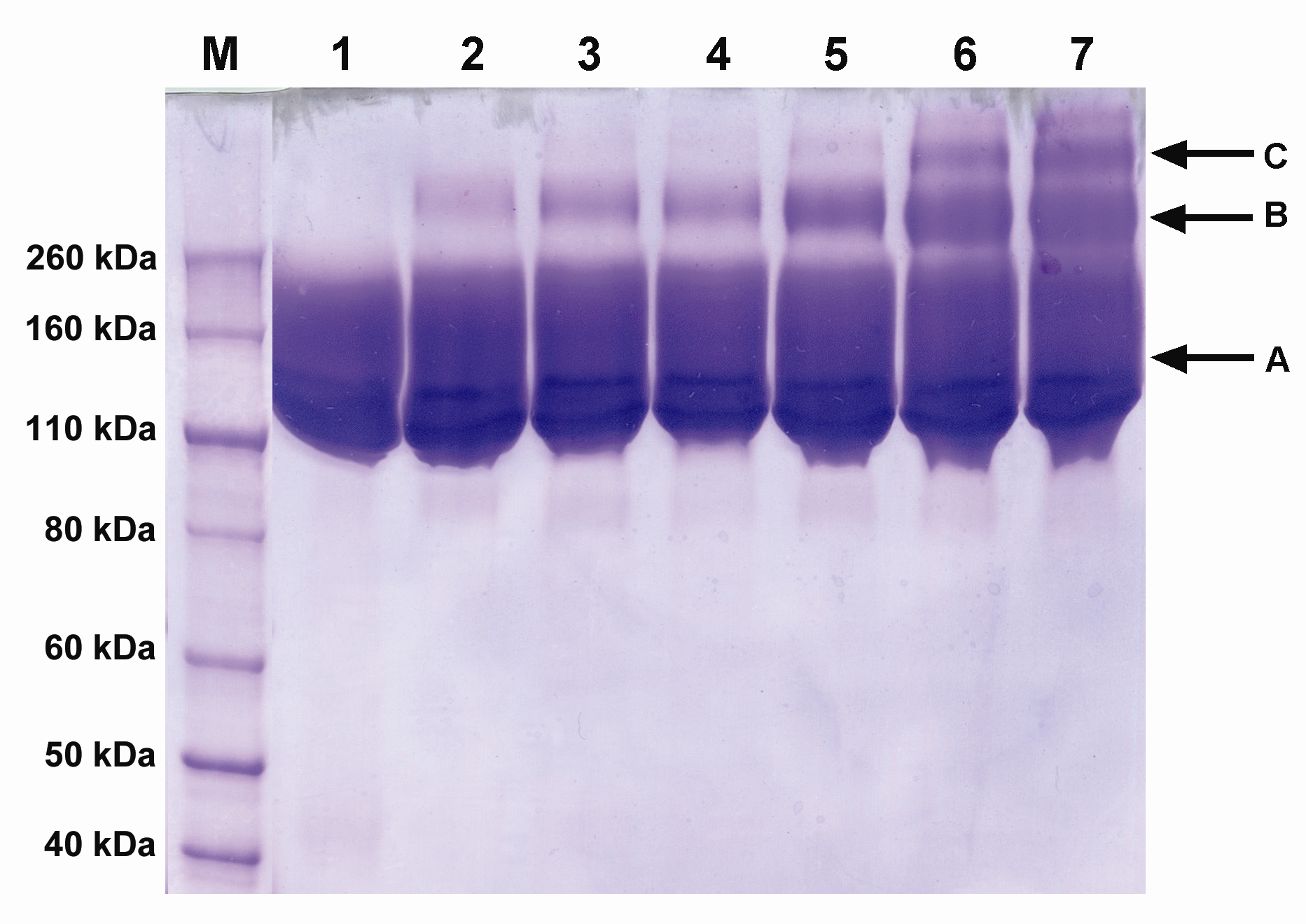


**SDS-PAGE analysis of mPEG-rCU with different contents of PEG diol**

Lanes 1 and 2 represent mPEG-rCU-1 and mPEG-rCU-2. Lanes 3-7 represent PEGylated rCU-1 with the different PEG contents: 0.5%, 1.0%, 1.5%, 2.0%, 2.7% (mPEG-rCU-3), respectively. A, B and C correspond to non-crosslinked pegylated monomeric rCU, crosslinked conjugates between two pegylated monomeric rCU and crosslinked conjugates among three monomeric rCU, respectively.
